# The Cow Milk Symptom Score (CoMiSS^TM^) in presumed healthy infants

**DOI:** 10.1371/journal.pone.0200603

**Published:** 2018-07-18

**Authors:** Yvan Vandenplas, Silvia Salvatore, Carmen Ribes-Koninckx, Eva Carvajal, Hania Szajewska, Koen Huysentruyt

**Affiliations:** 1 KidZ Health Castle, UZ Brussel, Vrije Universiteit Brussel, Brussels, Belgium; 2 Department of Pediatrics, University of Insubria, Ospedale “F. Del Ponte”, Varese, Italy; 3 Department of Pediatric Gastroenterology, Hepatology and Nutrition, La Fe University Hospital, Valencia, Spain; 4 Hospital Casa de Salud, Department of Paediatrics, Valencia, Spain; 5 Department of Paediatrics, the Medical University of Warsaw, Warsaw, Poland; Federal University of Sergipe, BRAZIL

## Abstract

**Objective:**

The Cow’s Milk-related Symptom Score (CoMiSS^TM^) was developed as an awareness tool to recognize possible manifestations of cow’s milk protein allergy (CMPA). Arbitrarily, a cut-off value of ≥12 was defined as a "positive score." The aim of this study was to determine an age-related CoMiSS in healthy infants to minimize the risk of false reassurance or over-diagnosis of CMPA in case of a negative or positive score, respectively.

**Methods:**

General pediatricians determined the CoMiSS in presumed healthy infants aged ≤6 months during a routine visit. Exclusion criteria included any known acute or chronic disease, preterm delivery (< 37 weeks), therapeutic formula, any food supplement (except vitamins) or medication.

**Results:**

Data from 891 consecutive infants were collected. Complete information was obtained from only 413 (46.4%) infants: Belgium: 31.2%, Italy 18.2%, Poland 19.1% and Spain 31.5%. Since gender (girls *vs* boys) (p = 0.579) had no influence on the CoMiSS, the data were re-calculated to include those infants with missing gender. The overall median and mean (SD) CoMiSS scores were, respectively, 3.0 and 3.7 (2.9). The 95th percentile was 9. Median crying (p<0.001), regurgitation (p = 0.009) and eczema (p = 0.039) scores differed significantly across the age categories. The other components of the CoMiSS were not age dependent.

**Conclusion:**

In healthy infants ≤ 6 months, the median CoMiSS is 3.0. More prospective studies in different sites and comparing healthy and allergic infants are warranted to obtain further evidence on the utility of the CoMiSS.

## Introduction

In 2015, a group of experts published a workshop report on the development of a Cow's Milk-related Symptom Score (CoMiSS^TM^) [1]. In the absence of specific diagnostic criteria for non-IgE cow’s milk protein (CMP) allergy (CMPA) and of predictive symptoms of response to a cow’s milk free diet, this score was proposed as an awareness tool for healthcare providers (HCPs) to increase the likelihood of attributing different signs and symptoms to the ingestion of cow’s milk [1]. The CoMiSS includes possible manifestations of CMPA such as gastrointestinal symptoms (regurgitation, altered stool composition), skin manifestations (eczema, urticaria), respiratory tract symptoms, and general symptoms such as crying time (Table A in S1 File). The CoMiSS ranges from 0 to 33 as complete absence of symptoms and signs to multiple and severe manifestations. Arbitrarily, by consensus of a panel of Belgian allergologists, pediatric gastroenterologists and general pediatricians, a cut-off value of ≥ 12 was proposed as a "positive score" to indicate the likelihood that the symptoms may be related to CMPA [2]. Several trials showed systematically a significant decrease in the score during an elimination diet and an increase during cow’s milk protein food challenge [2–5]. However, the score did not return to 0 in the responsive infants. As a consequence, the question did arise as to what the CoMiSS level would be in healthy infants and the influence of age on it. The goal of this study was to determine an age-related CoMiSS in healthy infants and to provide a scientific basis for a cut-off value with a low risk of false reassurance or over-diagnosis of CMPA by the tool.

## Methods

### Study design and data collection

This was an international multicenter cross-sectional study. General pediatricians from Belgium, Italy, Poland, and Spain determined a CoMiSS in presumed healthy infants ≤ 6 months of age. Participation was consecutively proposed to parents of "healthy" infants consulting for vaccination or a routine follow-up visit to monitor growth. Exclusion criteria were consulting the HCP because of any symptom or sign, and infants with known or suspected diseases such as neonatal complications, malformation, acute infection, known immune, neurologic, metabolic, cardiac, respiratory, endocrine or renal diseases, therapeutic formula such as an extensive hydrolysate formula, the administration of any food supplement (except the recommended vitamins) or medication, and preterm birth (<37 weeks). The following information was registered: gestational age, gender, age, and feeding type (breast milk or formula feeding), average crying time, regurgitation, stool consistency, skin and respiratory symptoms). The CoMiSS was registered anonymously, after informed consent from the parents. There was no funding for this study. Ethical approval from the corresponding ethical committees was obtained; the Ethical Committee of the UZ Brussel was the leading one.

### Statistical analysis

Statistical analysis was performed using the R statistical package (R version 3.1.2). A p value of <0.05 was considered statistically significant. Differences in proportions between groups were analyzed using a χ^2^-test or Fisher’s exact test, when appropriate. For continuous variables, the Mann Whitney U test or Kruskal-Wallis test was performed, when appropriate. Post hoc testing for skewed variables was done using a Nemenyi test. Since we expected an effect of age on most of the symptoms constituting the CoMiSS, the following a priori categorization for age was used for all age-related analyses: 0–1 month; 1–2 months, 2–3 months, 3–4 months, and 4–6 months. Based on the absence of gender influence on the CoMiSS in our population with complete data collection we further proceed to include infants with missing data on gender in the analysis handling this as a third variable.

## Results

### Population description

We recruited 891 infants. However, complete information was obtained from only 413 (46.2%) infants distributed as follows: Belgium: 129 (31.2%), Italy: 75 (18.2%), Poland: 79 (19.1%) and Spain 130 (31.5%). Data from Belgium were collected by 41 general pediatricians but were complete for only 129/566 (22.8%) infants. Data from Italy, Spain and Poland were collected by a selected group of HCPs (Italy n = 1, Spain n = 1, Poland n = 7) and were complete for all infants.

**Fig 1:**
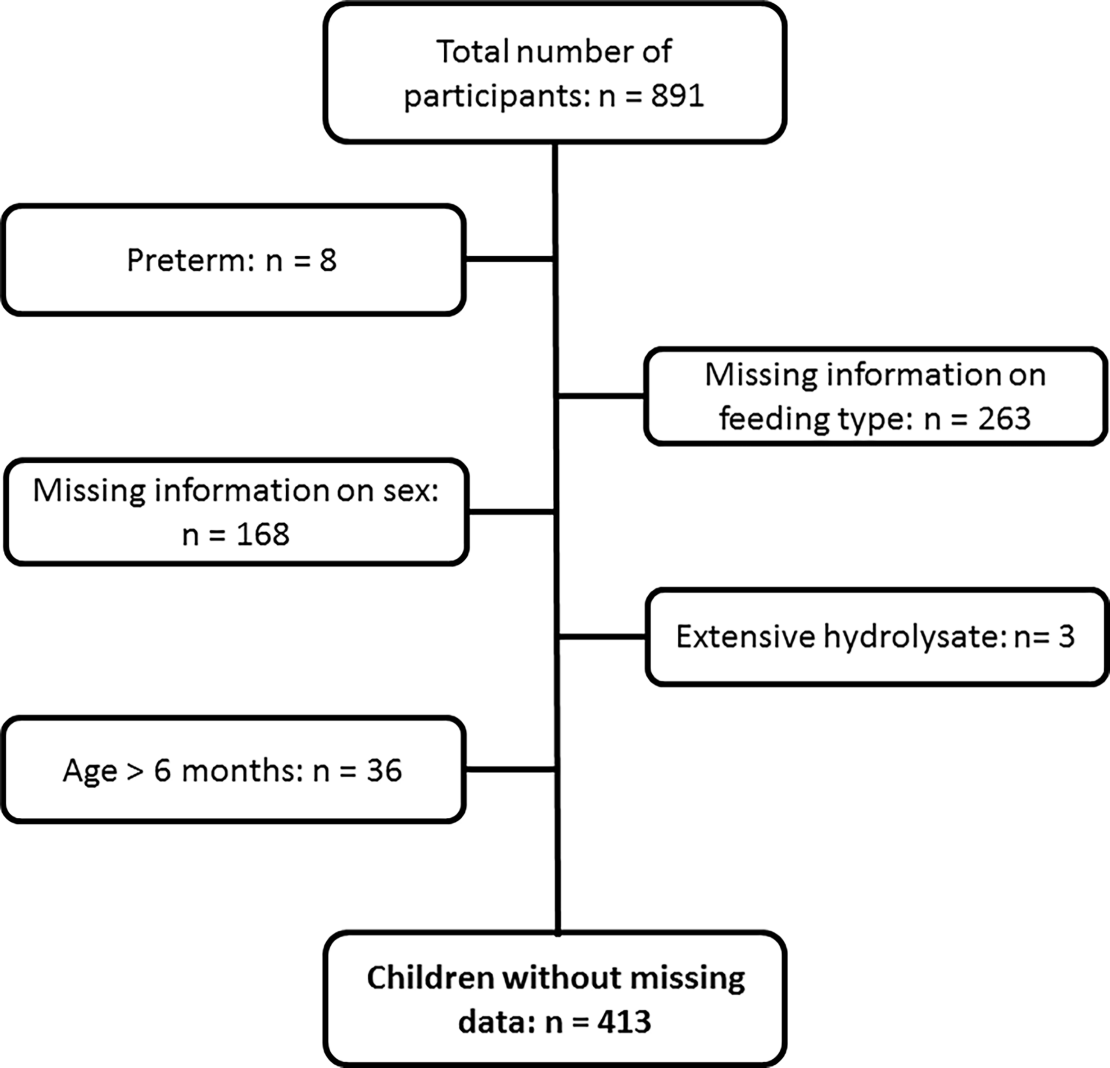
Participant flow chart.

A flowchart showing the number of participants including those with missing information is presented in Fig 1. Out of the 891 infants 263 were excluded from the study because information on feeding type was not reported and 65 did not comply with the inclusion/exclusion criteria. A flowchart of those with complete data is presented in the flowchart (Fig B in S2 File).

Patient demographic and feeding characteristics are presented in Table 1. Overall, 192 (34.1%) boys and 221 (39.3%) girls were included; data about gender were missing for 150 (26.6%) infants. A total of 283 (50.3%) infants were exclusively breastfed, 204 (36.2%) were exclusively formula fed, and 76 (13.5%) received mixed feeding. The overall median (Q1;Q3) age was 8.7 (4.1;16.0) weeks. Polish infants were significantly older than Belgian and Spanish infants (p<0.001) but not older than Italian infants (p = 0.40). Infants from Belgium were significantly younger than infants from Poland and Italy (p<0.001 for both countries) but not younger than Spanish infants (p = 0.49). There was no significant difference in median age between boys and girls (p = 0.41). The median age of those with a known gender (7.0 weeks) was significantly lower than for those with unknown gender (10.0 weeks; p = 0.02).

**Table 1:** Patient characteristics.

	Belgium N (%)	Italy N (%)	Poland N (%)	Spain N (%)	P value*
Total	279 (49.6)	75 (13.3)	79 (14.0)	130 (23.1)	
Boys	64 (22.9)	35 (46.7)	35 (44.3)	58 (44.6)	<0.001
Girls	65 (23.3)	40 (53.3)	44 (55.7)	72 (55.4)	
Unknown sex	150 (53.8)	0 (0)	0 (0)	0 (0)	
Exclusively breastfed	123 (44.1)	48 (64.0)	59 (74.7)	53 (40.8)	<0.001
Exclusively formula fed	122 (43.7)	21 (28.0)	11 (13.9)	50 (38.5)	
Mixed feeding	34 (12.2)	6 (8.0)	9 (11.4)	27 (20.8)	
Median age in weeks	7.0	12.0	14.4	8.9	<0.001^$^
(Q1;Q3)	(4.0;15.1)	(8.0;16.0)	(7.7;18.9)	(4.1;15.0)	
0–1 months	100 (35.8)	8 (10.7)	1 (1.3)	30 (23.1)	<0.001
1–2 months	64 (22.9)	19 (25.3)	20 (25.3)	26 (20.0)	
2–3 months	41 (14.7)	20 (26.7)	10 (12.7)	23 (17.7)	
3–4 months	31 (11.1)	16 (21.3)	20 (25.3)	21 (16.2)	
4–6 months	43 (15.4)	12 (16.0)	28 (35.4)	30 (23.1)	

*Χ^2^-test;

^$^Kruskal-Wallis test

### Overall results of the CoMiSS

**Fig 2:**
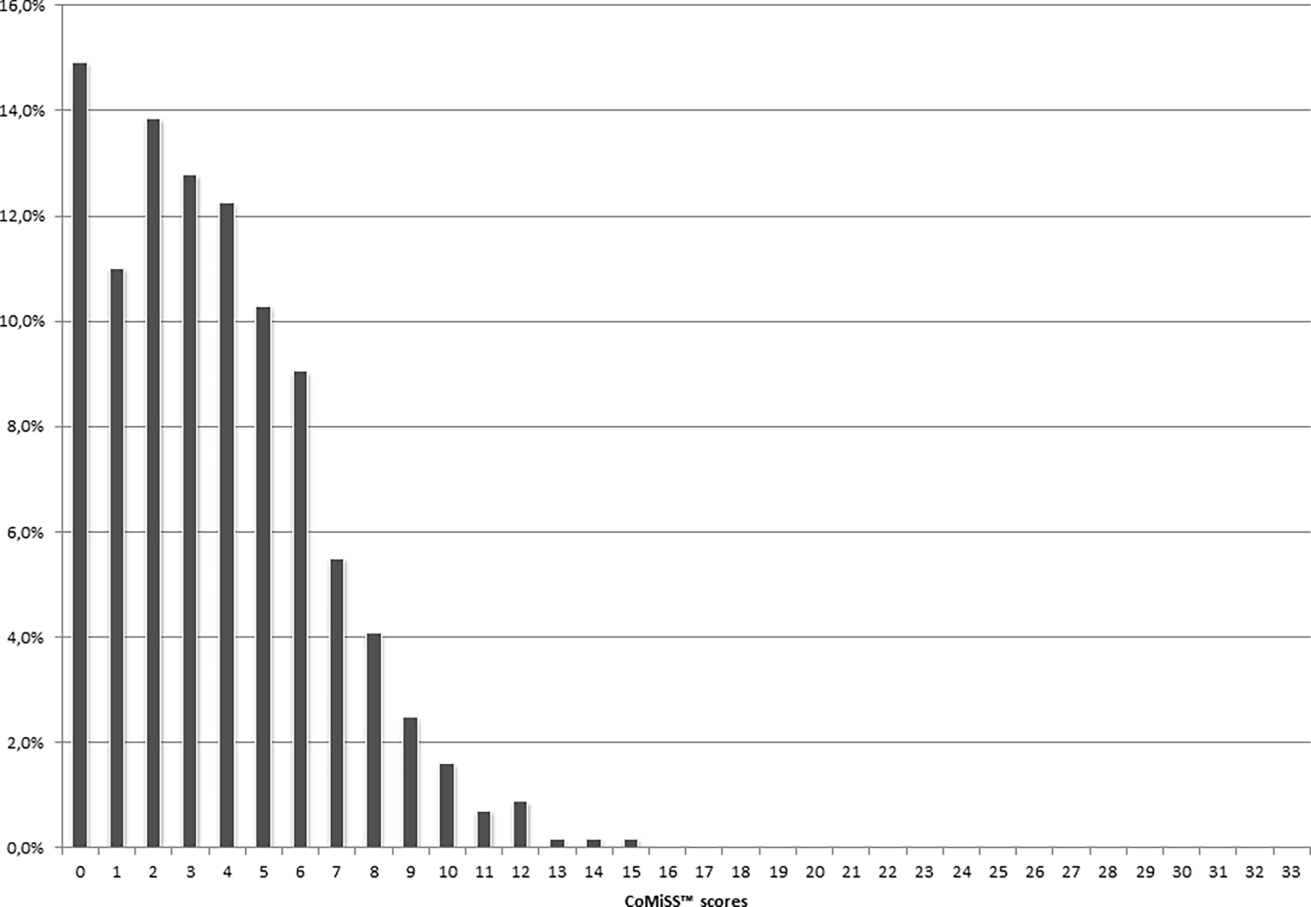
Distribution of the CoMiSS.

The distribution of the CoMiSS values is represented in Fig 2. The descriptive statistics for the CoMiSS are presented in detail in Table 2. The overall median and mean (SD) CoMiSS values were 3.0 and 3.7 (2.9), respectively. Only 1.5% of the infants had a CoMiSS ≥12, which was originally chosen by consensus as the arbitrary cut-off value for a “positive” score. The 95^th^ percentile for the CoMiSS for healthy infants was 9, and this finding was consistent among both genders and modes of feeding (**Table 2**). The 95^th^ percentile did, however, differ somewhat across countries and age categories, which can be attributed to the smaller groups in these sub-analyses.

**Table 2:** Descriptive statistics of the CoMiSS in healthy infants.

	P5	P25	Mean (SD)	Median	P75	P95	Min-max
Total (n = 563)	0	1	4 (3)	3	5	9	0–15
Boys (n = 192)	0	1	4 (3)	4	5	9	0–15
Girls (n = 221)	0	1	4 (3)	3	6	9	0–14
Belgium (n = 279)	0	1	3 (3)	3*	5	8	0–12
Italy (n = 75)	0	1	3 (3)	3*	6	8	0–11
Poland (n = 79)	1	3	5 (3)	4*	6	12	0–15
Spain (n = 130)	0	2	4 (3)	4*	6	9	0–11
Excl. breastfeeding (n = 283)	0	2	4 (3)	3	6	9	0–14
Non-excl. breastfeeding (n = 280)	0	1	4 (3)	3	5	9	0–15
< 1 month (n = 139)	0	1	3 (3)	3	5	8	0–10
1–2 months (n = 129)	0	2	4 (3)	4	6	10	0–14
2–3 months (n = 94)	0	2	4 (3)	3	6	9	0–10
3–4 months (n = 88)	0	1	4 (4)	4	6	11	0–15
4–6 months (n = 113)	0	1	3 (3)	3	5	8	0–12

*statistical difference across countries (p = 0.002)

**Fig 3:**
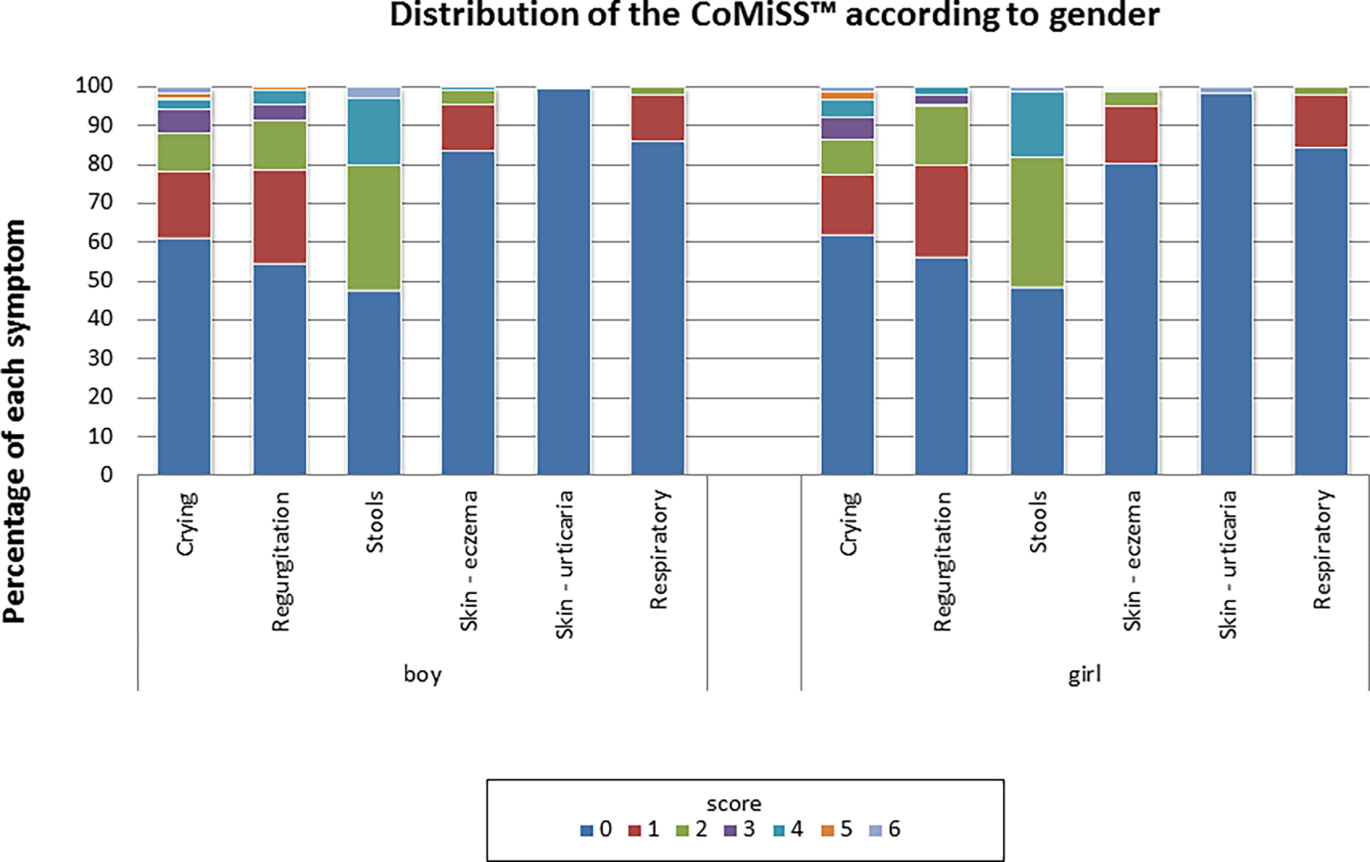
Distribution of CoMiSS according to gender.

**Fig 4:**
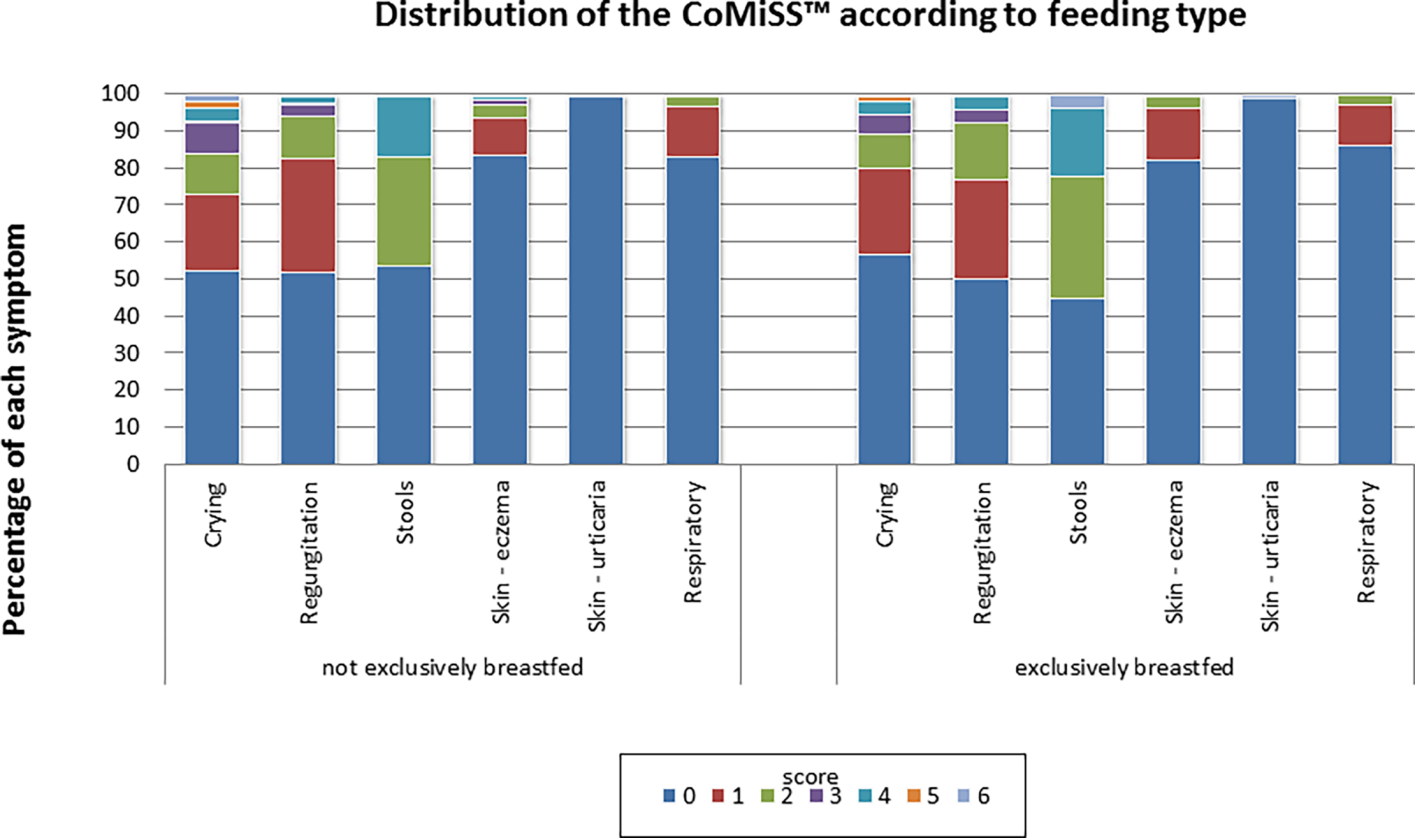
Distribution of CoMiSS according to feeding.

There was no significant difference in median CoMiSS value between groups based on gender (p = 0.76) **(**Fig 3) or feeding type (exclusively breastfed vs. non exclusively breastfed) (p = 0.46) (Fig 4). The high drop-out rate in Belgium was due to incomplete data registration of the feeding (Fig B in S2 File). Although feeding itself did not influence CoMiSS (Fig 4), we did not include the infants with "unknown feeding" as some of them may have been on a therapeutic formula. However, inclusion of these missing data would not have changed the outcome (data not shown). Age did influence the overall CoMiSS (Table 2; Fig C in S3 File). We found a statistical difference in median CoMiSS value across countries (p = 0.002). Post hoc testing revealed only a significant difference between Poland and Belgium (p = 0.001) and Poland and Italy (p = 0.02) as well as a trend toward a difference across age categories with higher values in the 1–2 months and 3–4 months age groups (p = 0.09 for overall difference, with post hoc test yielding p values <0.01 for each separate analysis (Table 2).

### Contribution of each symptom to the CoMiSS

**Fig 5:**
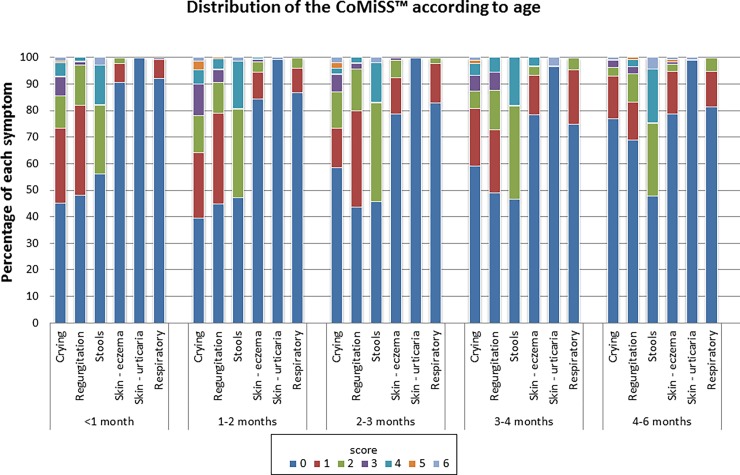
Distribution of CoMiSS according to age.

**Fig 6:**
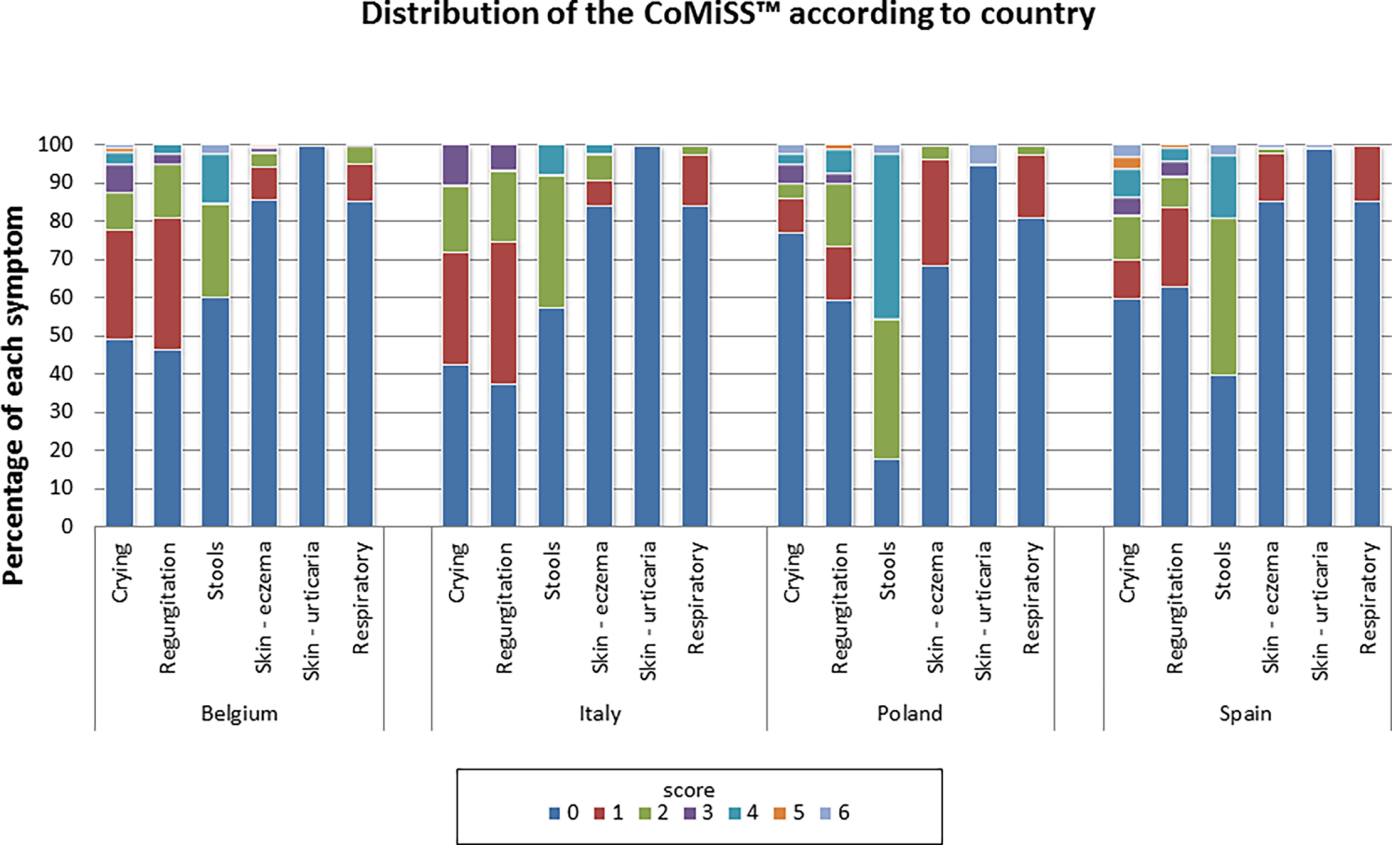
Distribution of CoMiSS according to country.

An overview of the contribution of each symptom to the CoMiSS is presented in Table D in S4 File. Crying scores are presented per country and per age class in Figs 5 and 6. Median crying scores were significantly different across the age categories (p<0.001) with decreasing time in older infants and reaching a score of 0 in 77% of the infants aged 4–6 months. Overall, only 36 (6.4%) of the infants cried >3 hours per day (score >3). And this occurred mainly in the younger age categories (36.1% in age category 1–2 months and 27.8% in category <1 month). In only 16 infants (2.8%) crying was reported to last more than 4 hours per day (CoMiSS crying score 5–6). The median crying scores were also significantly different across countries [p = 0.001; post hoc testing revealed significantly lower values for Polish infants vs. Belgian (p = 0.005), Italian (p = 0.006), and Spanish (trend: p = 0.06) infants]. However, half of the 36 infants that cried >3 hours/day were found in the Polish population, and none in Italy. There was no significant difference in median crying times based on gender (p = 0.98) or feeding type (p = 0.12).

Regurgitation scores are presented per country and per age class in Figs 5 and 6. Median regurgitation scores were significantly different across the age categories (p = 0.009). In total, 19 (3.4%)infants had a regurgitation score >3, which was mainly found in infants aged 1–2 months (6/19, 31.6%). The regurgitation score of 0 (less than two episodes of regurgitation per day) appeared less in the 2–3 months and more frequently in the 4–6 months age groups. No infant presented regurgitation of the total volume of feeding after each meal (CoMiSS regurgitation score 6) and in only two infants (0.4%) regurgitation of more than half the volume of feeding was reported in at least half meals (CoMiSS regurgitation score 5). Median regurgitation scores were also significantly different across countries (p = 0.01). None of the Italian infants had a score >3, but Italy also had the lowest percentage of children with a regurgitation score of 0 (37.3% vs. 46.6% of the Belgian infants, 59.5% of Polish infants, and 63.1% of Spanish infants). There was no significant difference in median regurgitation scores based on gender (p = 0.64) or feeding type (p = 0.36).

Stools were considered of normal or soft consistency (CoMiSS stool score 0–2) in 453 (80.5%) of the infants. The stool scores are presented per country and per age class in Figs 5 and 6. There was no significant difference between normal/soft stools *vs*. abnormal stools (score >2) across age categories (p = 0.61). The median stool score was significantly higher in infants exclusively breastfed compared to those not exclusively breastfed (2.0 vs. 0.0, p = 0.02). There was no significant difference in median stool scores based on gender (p = 0.717) or age category (p = 0.60). The median stool score in Poland was significantly higher than that in other countries (overall p<0.001).

Eczema was reported in 96/563 (17.1%) infants but only in 9 infants (1.6%) it was scored as moderate to severe (CoMiSS skin value ≥3). Surprisingly, urticaria was also reported, although in only 5/563 (0.9%) of healthy infants. The presence of skin symptoms is shown per country and per age class in Figs 5 and 6. Children without any sign of eczema were found most frequently in the lowest age categories (22.4% of those without eczema were <1 month and 19.4% between 1–2 months). The median eczema score was significantly different across age classes (p = 0.04) and country (p = 0.007), but post hoc testing yielded p values >0.10 for all comparisons among the different age categories. There was no difference in median eczema scores based on feeding type (p = 0.83) or gender (p = 0.41).

Only one infant had severe respiratory symptoms (CoMiSS respiratory score = 3); the vast majority of infants (476/563, 84.5%) did not show any respiratory symptom (score = 0). Respiratory scores are presented per country and per age class in Figs 5 and 6. There was no difference in median respiratory symptom scores across countries (p = 0.83), feeding type (p = 0.27), or gender (p = 0.62). However, the median respiratory symptom score did vary significantly across age classes (p = 0.008), with absence of symptoms (score 0) more frequently reported in the 0–1 months group, but post hoc testing did not show a significant difference for any of the comparisons among the different age categories (p values ≥0.19).

## Discussion

The median CoMiSS in healthy infants up to the age of 6 months among different countries was 3, and the 95^th^ percentile was 9. Overall, the CoMiSS was greater in the Polish infants (median = 4; 95^th^ percentile = 12) than in those from the other countries. This could be partially related to age difference since the Polish infants were older than those enrolled in Belgium, Italy and Spain. Since the CoMiSS does not differ between Italy, Spain and Belgium, it can be concluded that the score is not dependent on the training and information given to the HCPs, as in Spain and Italy the CoMiSS was determined by one investigator, compared to 41 general pediatricians in Belgium.

Overall, we found a weak trend for a higher CoMiSS with increasing age. Although the observation of a higher CoMiSS in Polish infants needs to be confirmed, it also suggests that parental perceptions and willingness to seek medical help may vary from country to country. Parents from Belgium, Italy, and Spain may consult HCPs more rapidly for (minor) healthcare issues related to their babies than parents in Poland, or HCP from those countries might more actively question parents for those minor issues. Functional gastrointestinal symptoms (e.g., regurgitation and consistency of stools), crying time, and eczema may be less often considered a medical problem for Polish parents resulting in the highest CoMiSS in Polish infants. This hypothesis is illustrated by the fact that 50% of the infants that cried for > 3 hours were from Poland. The sooner parents seek medical help, the more HCPs will intervene. The market share of therapeutic formula may be considered as an indirect indicator. The differences in crying time according to age confirm the age-related differences in durations of crying times reported previously in the literature [6]. Our findings also suggest that half of healthy infants < 6 months of age do regurgitate, confirming data from the literature [7]. We observed no difference in this symptom between breast- and formula- fed infants despite the previous report that exclusively breastfed infants regurgitate less than partially breastfed infants [8]. The differences in stool scores based on feeding type can be explained by a CoMiSS stool value of 4 indicating both watery stools, that can normally occur in healthy breastfed babies, and hard stools. Interestingly, the incidence and severity of atopic dermatitis did not differ according to feeding type, suggesting that mild to moderate eczema may be less frequently related to cow’s milk protein than often considered [9]. This finding also needs to be confirmed. Respiratory symptoms were seldom reported, and were more prevalent with increasing age, as could be expected.

Depending on age, the lowest P95 value was 8 and the highest was 11. Median scores were 3 and 4 and P75 values were 5 and 6, varying with age. Our findings in healthy infants endorse the high prevalence of a positive oral food challenge test (Odds ratio 0.83; 95% confidence interval, 0.75–0.93) if a cut-off value of ≥ 12 is used, as previously reported [10]. As unrecognized cow’s milk allergy does have a long term negative health impact, under-diagnosis should be avoided whenever possible [11,12]. Over-diagnosis of CMPA would result in unneeded dietary interventions, and higher economic health costs but without a major negative health impact. Therefore, further studies should be performed to determine the optimal cut-off value in symptomatic infants. Since a CoMiSS of 6 reflects the highest P75 value, ≤ 6 seems to be a useful cut-off value to document effective treatment of cow’s milk-related symptoms in symptomatic infants. Hence, the results of our study provide useful information for interpretation of the evolution of the CoMiSS in infants suspected of presenting with cow’s milk-related symptoms during a dietary elimination diet. However, the CoMiSS is an awareness tool and not a diagnostic tool for CMPA; an elimination diet followed by a challenge test remains required to diagnose CMPA.

A limitation of our study is that the information obtained by the HCPs was based on a retrospective recall by the parents at the moment of the consult. It is well known that retrospective recall of symptoms results in a trend towards an increased perception compared to a prospective collection in a diary e.g. during a period of three days [13]. However, in daily life, this information is obtained as a recall during a consultation. The high drop-out rate in the Belgian infants because of incomplete information is another limitation. However, since the data obtained from Belgium do not differ from those from Spain and Italy, it illustrates that CoMiSS scores do not depend on the training of the investigator.

## In conclusion

The CoMiSS was determined in a large series of healthy infants, resulting in a median of 3, mean of 3.85 and P95 of 9. These data can be helpful for the HCP to support the diagnosis and management of infants presenting with multiple gastrointestinal, respiratory and skin symptoms suspected of functional gastrointestinal disorders or CMPA. Infants with a high CoMiSS are symptomatic infants and should undergo exclusion diet and CMP challenge to confirm the diagnosis of CMPA. More prospective studies in different sites and comparing healthy and allergic infants are indicated to obtain further evidence on the utility of the CoMiSS.

## Supporting information

S1 File Table A. Composition of the CoMiSS.
(DOCX)

S2 File Figure B. Flow chart of children without any missing data.
(TIF)

S3 File Figure C. Age distribution of the CoMiSS.
(TIF)

S4 File Table D. Contribution of each symptom to the CoMiSS.
(DOCX)
