# Supplementary material for: The Cow Milk Symptom Score (CoMiSSTM) in presumed healthy infants
Source: PLoS One. 2018 Jul 18;13(7):e0200603. doi: 10.1371/journal.pone.0200603 (PMC6051613; doi:10.1371/journal.pone.0200603)
Supplement: S1 File — Table A. Composition of the CoMiSS. (DOCX) [file pone.0200603.s001.docx]

**Supplementary File 1:** CoMiSS^TM^

| Symptom | Score |  |
| --- | --- | --- |
| Crying (°) | 0  1  2  3  4  5  6 | < 1 hour/day  1 - 1.5 hours/day  1.5 - 2 hours/day  2 to 3 hours/day  3 to 4 hours/day  4 to 5 hours/day  > 5 hours/day |
| Regurgitation | 0  1  2  3  4  5  6 | 0-2 episodes/day  > 3 - < 5 of small volume  > 5 episodes of > 1 coffee spoon  > 5 episodes of + half of the feed in < half of the feeds  continuous regurgitations of small volumes > 30 min after each feed  regurgitation of half to complete volume of a feed in at least half of the feeds  regurgitation of the complete feed after each feeding |
| Stools  (Bristol scale) | 4  0  2  4  6 | type 1 and 2 (hard stools)  type 3 and 4 (normal stools)  type 5 (soft stool)  type 6 (liquid stool, if unrelated to infection)  type 7 (watery stools) |
| Skin symptoms | 0 to 6  0 or 6 | Atopic eczema  Head neck trunk Arms hands legs feet  Absent 0 0  Mild 1 1  Moderate 2 2  Severe 3 3  Urticaria (no 0 / yes 6) |
| Respiratory symptoms | 0  1  2  3 | no respiratory symptoms  slight symptoms  mild symptoms  severe symptoms |

***Legend***

(§) Although many infants with cow's milk related symptoms have no impaired growth or weight gain, faltering of these parameters suggests organic disease, of which CMPA is a possible cause.

(°) Crying was only considered if the child was crying for one week or more, assessed by the parents, without any other obvious cause.
