# Supplementary figures and images for: The Cow Milk Symptom Score (CoMiSSTM) in presumed healthy infants
Source: PLoS One. 2018 Jul 18;13(7):e0200603. doi: 10.1371/journal.pone.0200603 (PMC6051613; doi:10.1371/journal.pone.0200603)

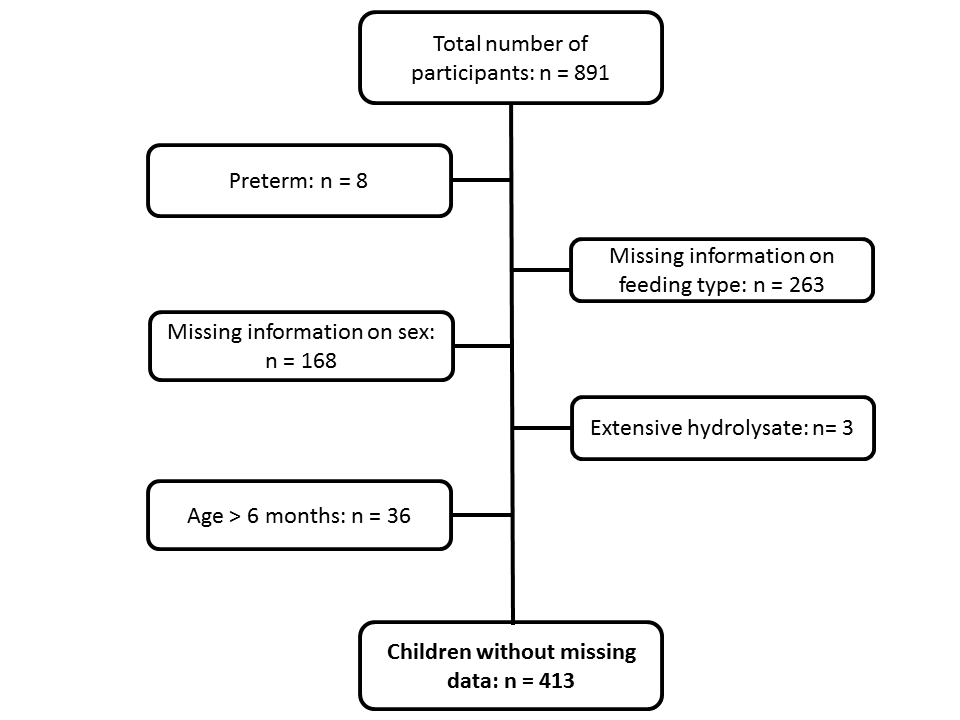

Supplement: S2 File — Figure B. Flow chart of children without any missing data. (TIF) [file pone.0200603.s002.tif]

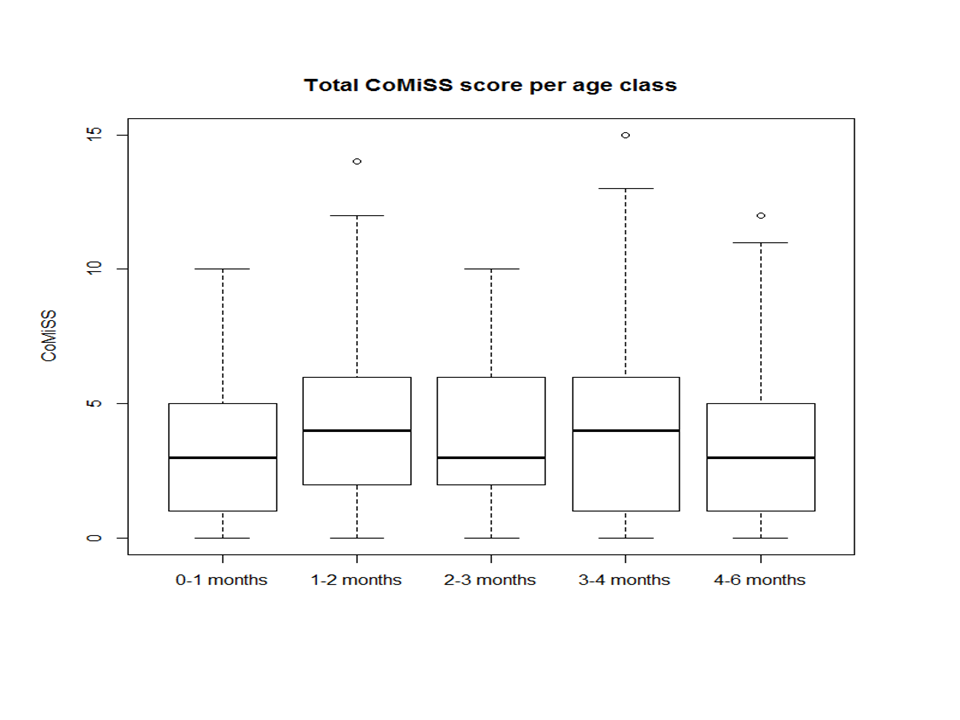

Supplement: S3 File — Figure C. Age distribution of the CoMiSS. (TIF) [file pone.0200603.s003.tif]
