# Supplementary material for: The Cow Milk Symptom Score (CoMiSSTM) in presumed healthy infants
Source: PLoS One. 2018 Jul 18;13(7):e0200603. doi: 10.1371/journal.pone.0200603 (PMC6051613; doi:10.1371/journal.pone.0200603)
Supplement: S4 File — Table D. Contribution of each symptom to the CoMiSS. (DOCX) [file pone.0200603.s004.docx]

**Supplementary File D** Contribution of each symptom to the CoMiSS

| **Symptom** | **Score** | **N (%)** |
| --- | --- | --- |
| Crying | 0 | 308 (54.7) |
|  | 1 | 122 (21.7) |
|  | 2 | 58 (10.3) |
|  | 3 | 39 (6.9) |
|  | 4 | 20 (3.6) |
|  | 5 | 8 (1.4) |
|  | 6 | 8 (1.4) |
| Regurgitation | 0 | 287 (51.0) |
|  | 1 | 162 (28.8) |
|  | 2 | 76 (13.5) |
|  | 3 | 19 (3.4) |
|  | 4 | 17 (3.0) |
|  | 5 | 2 (0.4) |
|  | 6 | 0 (0) |
| Stools | 0 | 277 (49.2) |
|  | 2 | 176 (31.3) |
|  | 4 | 97 (17.2) |
|  | 6 | 13 (2.3) |
| Skin - eczema | 0 | 467 (82.9) |
|  | 1 | 67 (11.9) |
|  | 2 | 20 (3.6) |
|  | 3 | 3 (0.5) |
|  | 4 | 4 (0.7) |
|  | 5 | 1 (0.2) |
|  | 6 | 1 (0.2) |
| Skin - urticaria | 0 | 558 (99.1) |
|  | 6 | 5 (0.9) |
| Respiratory | 0 | 476 (84.5) |
|  | 1 | 69 (12.3) |
|  | 2 | 17 (3.0) |
|  | 3 | 1 (0.2) |
